# Omission of Throat Anesthesia Using Jackson’s Spray Prior to Bronchoscopy for Preventing Aerosol Generation: A Survey Through Patient Distress Questionnaire

**DOI:** 10.7759/cureus.17231

**Published:** 2021-08-16

**Authors:** Saori Murata, Shinji Sasada, Yusuke Usui, Tetsuya Sakai, Keisuke Kirita, Kota Ishioka, Saeko Takahashi, Morio Nakamura

**Affiliations:** 1 Department of Pulmonary Medicine, Tokyo Saiseikai Central Hospital, Tokyo, JPN; 2 Department of Thoracic Oncology, National Cancer Center Hospital East, Kashiwa, JPN

**Keywords:** throat anesthesia, jackson’s spray, bronchoscopy, questionnaire, aerosol, covid-19

## Abstract

Background and objective

Due to the outbreak of coronavirus disease 2019 (COVID-19), the Japanese Society of Respiratory Endoscopy recommended the omission of throat anesthesia using Jackson’s spray prior to bronchoscopy for preventing aerosol generation. In this survey, we investigated the tolerability of patients toward the omission of anesthesia using Jackson’s spray before bronchoscopy.

Methods

Group A patients received throat anesthesia with 5 mL of 4% lidocaine using Jackson’s spray prior to bronchoscopy and were then administered pethidine hydrochloride and midazolam intravenously. Group B patients did not receive anesthesia using Jackson’s spray before bronchoscopy. They were administered pethidine hydrochloride and midazolam and were then administered 8% lidocaine several times into the pharynx. A patient distress questionnaire, classified as a five-graded score, was administered to each group after bronchoscopy.

Results

Seventy patients participated in this study: 39 patients in Group A and 31 patients in Group B. There were no significant differences in their backgrounds. In the questionnaire survey, the distress caused by pre-examination anesthesia in Group A was significantly higher than in Group B (3.03 ± 1.25 vs. 1.23 ± 0.62; *p* < 0.0001), and no significant differences were observed in the other questions during bronchoscopy.

Conclusion

This study demonstrates the tolerability of patients toward the omission of throat anesthesia using Jackson’s spray prior to bronchoscopy, which is recommended for preventing infection, including COVID-19.

## Introduction

Lidocaine spray is recommended as a conventional method of throat anesthesia during bronchoscopy by the British Thoracic Society (BTS) and the American College of Chest Physicians (ACCP) [1,2]. In Japan, Jackson’s spray has been widely used to administer throat anesthesia before bronchoscopy [3]. However, it has been reported that patients were more distressed by throat anesthesia than by the examination itself [4]. In 2020, the pandemic of coronavirus disease 2019 (COVID-19), whose etiologic agent is severe acute respiratory syndrome coronavirus 2 (SARS-CoV-2), swept the world. The novel coronavirus is believed to be transmitted through aerosols. Accordingly, the Japanese Society of Respiratory Endoscopy recommended the omission of throat spray anesthesia before bronchoscopy to prevent aerosol generation. However, it is unclear if the pain during bronchoscopy is consequently exacerbated. Therefore, we administered a patient distress questionnaire examining the tolerability toward the omission of throat spray anesthesia prior to bronchoscopy, historically compared with the conventional procedure.

## Materials and methods

Subjects

The study design was a prospective observational study (Group B), which was compared with past data (Group A). Group A consisted of patients who underwent bronchoscopy at Tokyo Saiseikai Central Hospital via conventional methods between August 2016 and December 2016. In this group, throat anesthesia using Jackson’s spray was administrated prior to bronchoscopy, and sedatives were administered intravenously. Group B consisted of patients who underwent bronchoscopy between May 2020 and November 2020. In this group, throat spray anesthesia was omitted before bronchoscopy. All the examinations were standby bronchoscopies; emergency and bedside bronchoscopies were excluded from this study. The questionnaire was obtained consecutively in each period. It was approved by the Clinical Research Ethics Committee of Saiseikai Central Hospital and was implemented (approval number 2020-010-1).

Procedure

**Figure 1:**
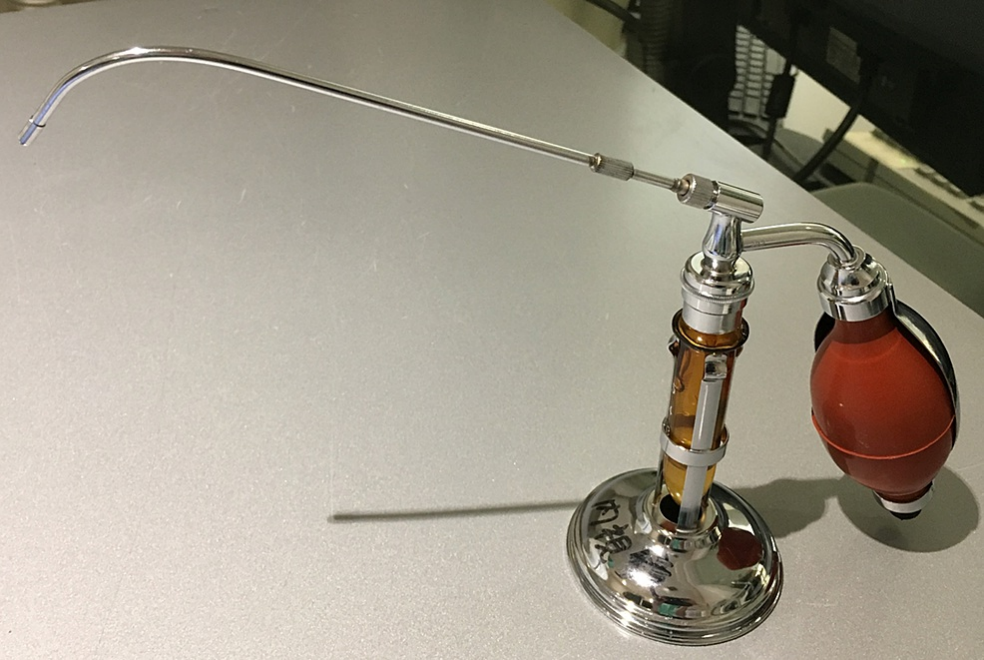
Jackson’s spray

**Figure 2:**
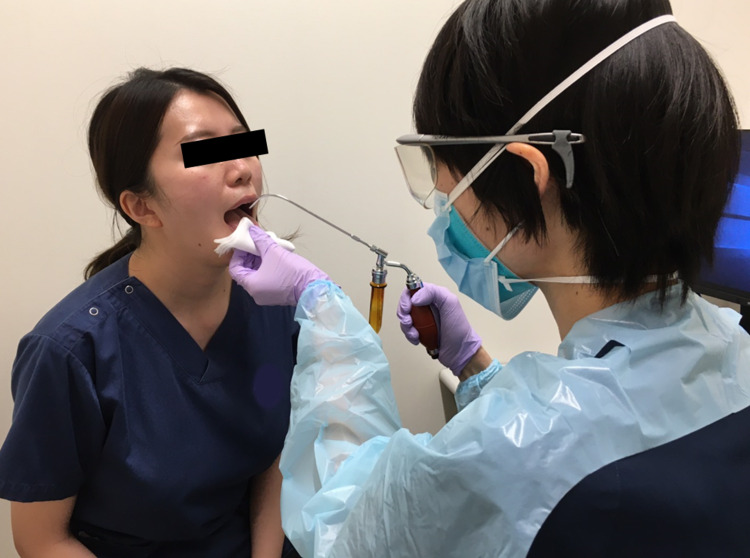
Throat anesthesia using Jackson’s spray Five milliliters of 4% lidocaine was administered face-to-face prior to bronchoscopy.

In Group A, throat anesthesia of 5 mL of 4% lidocaine using Jackson’s spray was administered face-to-face prior to bronchoscopy (Figures 1, 2), and 17.5 mg of pethidine hydrochloride and 3 mg of midazolam were administered intravenously.

In Group B, throat spray anesthesia was omitted before bronchoscopy. The patients received 17.5 mg of pethidine hydrochloride and 3 mg of midazolam followed by 8% lidocaine several times into the pharynx. In both groups, the bronchoscope was inserted orally, and 10 mL of 2% lidocaine was applied to each bronchus via the working channel of the bronchoscope. In case of frequent coughing, additional doses of pethidine (17.5 mg) or midazolam (1-2 mg) were administered at the discretion of the operator. The initial dose of midazolam was reduced to 2 mg in patients aged 80 years or older or in patients with a bodyweight of 45 kg or lower. In addition, a nasal cannula with 3 L/min of oxygen was routinely administered. Flexible bronchoscopy was performed using either BF-1T260 (outer diameter of 5.9 mm, Olympus, Tokyo, Japan), BF-1TQ290 (outer diameter of 5.9 mm, Olympus, Tokyo, Japan), or BF-UC260FW (outer diameter of 6.9 mm, Olympus, Tokyo, Japan). The operator chose a bronchoscope considering the purpose of examination and location of the lesion.

Patient distress questionnaire

The questionnaire used to evaluate the level of patient distress during bronchoscopy was based on a national questionnaire conducted by the Japan Society for Respiratory Endoscopy [5]. A five-graded score was used to evaluate the level of distress caused by the spraying of chemicals into the pharynx before the examination, in terms of the level of distress during the examination, the presence or absence of the memory of the examination, pain during the examination, the realization of examination time, and consent to retesting. Score 1 indicated the best result, and score 5 indicated the worst result. The questionnaire was administered more than two hours after bronchoscopy when the patients were fully awake.

Statistical analysis

Categorical variables, such as patient characteristics and procedures, were described using percentages, medians, and ranges for quantitative variables. All the statistical analyses were performed using EZR (Saitama Medical Center, Jichi Medical University, Saitama, Japan). Fisher’s exact test was used to analyze whether there was a statistically significant association between the two variables. In unpaired data, the differences were compared using the Student’s two-tailed t-test. A significance level of *p* < 0.01 was considered statistically significant in all the statistical tests.

## Results

Seventy patients participated in this study: 39 patients in Group A and 31 patients in Group B. Their characteristics are listed in Table 1.

**Table 1:** Patient characteristics SE, standard deviation; SpO2, saturation of peripheral oxygen; BP, blood pressure, *Fisher’s exact test.

Patient characteristics		All cases, N = 70		
Age, years, median (range)		71.5 (19-89)		
Sex, number of males (%)		43 (61.4)		
	Group A, N = 39 (Jackson’s spray)		Group B, N = 31 (no spray)	*p*-value*
Age, years, median (range)	73 (19-89)		67 (26-84)	0.49
Sex, number of males (%)	24 (61.5)		19 (61.3)	1.00
Examination time, min, median (range)	20 (6-75)		27 (7-56)	0.09
Midazolam dose, mg, mean ± 2SE	3.4 ± 1.1		3.7±1.4	0.27
Pethidine dose, mg, mean ± 2SE	17.5 ± 0		22.6±8.1	<0.001
Baseline SpO2, %, mean ± 2SE	98 ± 2.2		98 ± 1.3	0.14
Minimum SpO2, %, mean ± 2SE	95 ± 3.7		94 ± 7.7	0.41
Change of SpO2, %, mean ± 2SE	2.8 ± 3.2		4.7 ± 7.3	0.17
Oxygen dose, L/min, mean ± 2SE	3.8 ± 1.6		4.0 ± 1.9	0.61
Baseline systolic BP, mmHg, mean ± 2SE	140 ± 20		142 ± 25	0.74
Minimum systolic BP, mmHg, mean ± 2SE	118 ± 19		129 ± 26	0.02

Their median age was 71.5 years (range, 19-89 years), and 43 of them (61.4%) were men. There were no significant differences in sex, age, or examination time between the two groups. The mean doses of pethidine hydrochloride in Group A were significantly lower than those in Group B (17.5 ± 0 vs. 22.6 ± 8.1, *p* < 0.001). The baseline and changes in the hemodynamic monitoring parameters (oxygen saturation, oxygen dose, and blood pressure) were not significantly different between the two groups. The frequency of bronchoscopy procedures (observation, bronchial alveolar lavage [BAL], transbronchial biopsy [TBB] or transbronchial lung biopsy [TBLB] with endobronchial ultrasound through a guide sheath [EBUS-GS], TBB or TBLB without EBUS-GS, and endobronchial ultrasound-guided transbronchial needle aspiration [EBUS-TBNA]) and the selection of bronchoscopes were not significantly different between the two groups (Table 2).

**Table 2:** Bronchoscopy procedure and selection of bronchoscope BAL, bronchial alveolar lavage; TB(L)B, transbronchial (lung) biopsy; EBUS-GS, endobronchial ultrasound through a guide sheath, EBUS-TBNA: endobronchial ultrasound-guided transbronchial needle aspiration, *Fisher’s exact test.

Procedures	Group A, N = 39 (Jackson’s spray)	Group B, N = 31 (no spray)	*p*-value*
Observations	2	1	1.00
BAL	7	12	0.060
TB(L)B with EBUS-GS	13	13	0.62
TB(L)B without EBUS-GS	7	5	1.00
EBUS-TBNA	12	13	0.45
Others	1	0	1.00
Bronchoscope			
BF-1T260	29	18	0.58
BF-1TQ290	0	7	
BF-UC260FW	10	13	0.20

**Figure 3:**
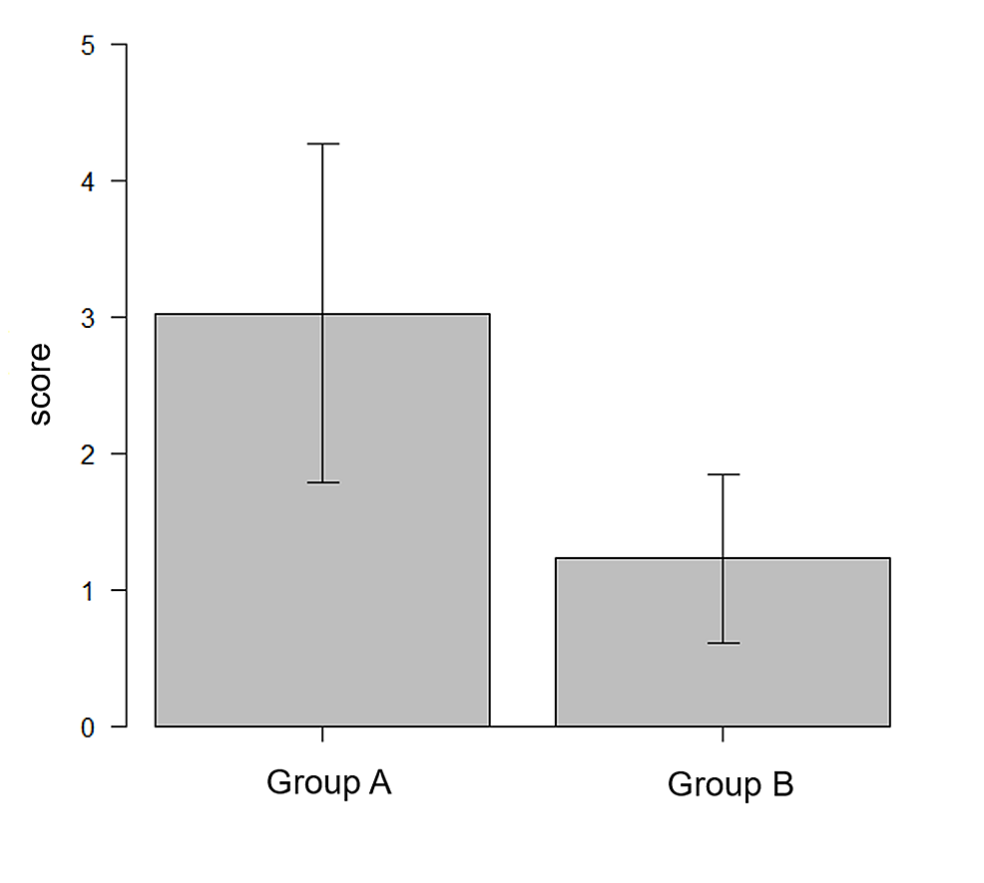
Patient distress caused by pre-examination anesthesia The score of Group A was significantly higher than that of Group B (3.03 ± 1.25 vs. 1.23 ± 0.62; *p *< 0.0001, Student’s two-tailed t-test). Scores were graded between 1 (best) and 5 (worst).

From the results of the questionnaire, the distress caused by preoperative anesthesia in Group A was significantly higher than that in Group B (3.03 ± 1.25 vs. 1.23 ± 0.62; *p* < 0.0001) (Figure 3).

On the other hand, the patient distress during bronchoscopy, in terms of discomfort during the examination, the memory of the examination, pain during the examination, examination duration, and consent to re-examination, was not significantly different between the two groups (Table 3).

**Table 3:** Patient distress during bronchoscopy Scores were graded from 1 (best) to 5 (worst). *Student’s two-tailed t-test; ‡asthma attack.

Question items	Group A, N = 39 (Jackson’s spray)	Group B, N = 31 (no spray)	*p*-value*
Discomfort during examination	1.56 ± 1.12	1.84 ± 1.29	0.35
Memory of examination	1.82 ± 1.23	2.29 ± 1.64	0.18
Pain during examination	1.23 ± 0.74	1.29 ± 0.82	0.75
Examination duration	1.56 ± 0.97	1.58 ± 1.06	0.95
Consent to re-examination	1.90 ± 1.23	1.48 ± 1.06	0.14
Complication	0	1^‡^	1.00

Complications occurred in one patient in Group B who could not complete the examination due to an asthma attack.

## Discussion

This study indicates that omission of throat anesthesia using Jackson’s spray prior to bronchoscopy provides reducing the patient distress during bronchoscopy (3.03 ± 1.25 vs. 1.23 ± 0.62; *p *< 0.0001). The BTS guidelines recommend the use of a combination of sedative drugs, particularly midazolam, and the use of lidocaine for local anesthesia. There are no absolutes in the combination of sedatives, and there is no consensus regarding the order of administration or the procedure for local anesthesia [1]. Usuda et al. showed that patients who received nebulized lidocaine for pharyngeal anesthesia followed by intravenous midazolam and a 1% lidocaine spray were less distressed than those who received a 1% lidocaine spray before midazolam administration. This indicates that administering a sedative prior to local anesthesia alleviates patient distress [6]. However, nebulization with lidocaine demonstrated no benefit, and it was not recommended in the BTS guidelines [7]. This procedure requires further improvement. The COVID-19 pandemic began at the end of December 2019. The novel coronavirus is believed to be transmitted through aerosols [8], and the prevention of aerosol exposure among medical staff during endoscopy is essential. Throat anesthesia using Jackson’s spray is a conventional method used in Japan. The patient sits in a chair, and the operator sits face to face, lightly pinches the tongue, inserts the tip of the spray into the mouth, and sprays into the laryngopharynx. As most patients cough due to irritation, there is a high risk of aerosol generation, and a previous report has shown that throat spray anesthesia is considerably painful [4].

In our study, Group A demonstrated significantly higher scores for discomfort due to pre-examination anesthesia, but other questions, such as distress before or during examination, presence or absence of memory of examination, pain during the examination, the realization of examination time, and consent to retesting, were comparable between the two groups. In other words, an omission of throat spray anesthesia did not increase the distress during bronchoscopy. However, more amount of pethidine hydrochloride was used in Group B, which may indicate the result of a high frequency of coughing during bronchoscopy. According to the results, wearing a face mask always must be considered by medical staff during the procedure.

The limitation of this study is the small number of participants in a single facility. Another limitation is that the choice of scope and procedure was at the discretion of the attending physician, and there may be a case selection bias. Moreover, the effect of the omission of throat spray anesthesia on the intensity of coughing is unclear. Further investigation through randomized controlled trials with a larger number of patients using the same method of sedation and analgesia is required.

## Conclusions

This study demonstrated the patient’s tolerability toward the omission of throat anesthesia using Jackson’s spray prior to bronchoscopy, which is recommended for preventing infection, including COVID-19.
